# Impact of sex on treatment-related adverse effects and prognosis in nasopharyngeal carcinoma

**DOI:** 10.1186/s12885-023-11564-0

**Published:** 2023-11-25

**Authors:** Linchong Cui, Zilu Chen, Fangfang Zeng, Xiaolan Jiang, Xiaoyan Han, Xiaofei Yuan, Shuting Wu, Huiru Feng, Danfan Lin, Wenxuan Lu, Xiong Liu, Xiaohong Peng, Bolong Yu

**Affiliations:** Department of Otolaryngology-Head and Neck Surgery, Nanfang Hospital, Southern Medical University, Guangzhou, 510515 Guangdong PR China

**Keywords:** Nasopharyngeal carcinoma, Adverse effects, Sex, Menopause, Chemotherapy

## Abstract

**Background:**

In nasopharyngeal cancer (NPC), women have a lower incidence and mortality rate than men. Whether sex influences the prognosis of NPC patients remains debatable. We retrospectively examined the influence of sex on treatment-related side effects and prognosis in NPC.

**Methods:**

Clinical data of 1,462 patients with NPC treated at the Southern Hospital of Southern Medical University from January 2004 to December 2015 were retrospectively examined. Statistical analysis was performed to assess differences in overall survival (OS), distant metastasis-free survival (DMFS), local recurrence-free survival(LRFS), and progression-free survival(PFS), as well as treatment-related adverse effects, including myelosuppression, gastrointestinal responses, and radiation pharyngitis and dermatitis, between men and women.

**Results:**

Women had better 5-year OS (81.5% vs. 87.1%, *P* = 0.032) and DMFS (76.2% vs. 83.9%, *P* = 0.004) than men. Analysis by age showed that the prognoses of premenopausal and menopausal women were better than those of men, whereas prognoses of postmenopausal women and men were not significantly different. Additionally, women had a better prognosis when stratified by treatment regimen. Furthermore, chemotherapy-related adverse effects were more severe in women than in men; however, the incidences of radiation laryngitis and dermatitis were not significantly different between the sexes. Logistic regression analysis revealed that the female sex was an independent risk factor for severe myelosuppression and gastrointestinal reactions.

**Conclusions:**

Chemotherapy-related side effects are more severe but the overall prognosis is better in women with NPC than in men with NPC. Patients may benefit from a personalized treatment approach for NPC.

**Trial registration:**

This study was approved by the Medical Ethics Committee of Nanfang Hospital of the Southern Medical University (NFEC-201,710-K3).

## Introduction

Nasopharyngeal carcinoma (NPC), a common malignancy of the head and neck region, has notable regional and sexual differences. More than 70% of all new cases have been diagnosed in East and Southeast Asia. The incidence of NPC is approximately 2.75 times higher in men than in women, according to the latest 2021 Cancer Journal for Clinicians [1]. Smoking, drinking, occupational risk factors, and other characteristics may contribute to the higher incidence in men [2]. A previous study suggested that sex hormones may play a role in the disparity between sexes [3].

In addition to pathological types, TNM stages, and treatment regimens [4, 5], sex has a significant impact on the prognosis of NPC patients, which may be related to the inherent differences between sexes, especially the levels of sex hormones [6–9]. Aside from prognosis, sex also influences the occurrence of adverse reactions in the treatment of malignant tumors. Due to the high toxicity of antineoplastic drugs, adverse reactions often occur in the treatment of malignant tumors. Existing research has discovered that in the treatment of non-reproductive organ cancers (such as lung cancer [10],digestive tract tumors [11], gliomas [12], and lymphomas [13]), the incidence and severity of adverse reactions, including hematotoxicity, nausea, vomiting, oral ulcers, and hair loss, were higher in women than in men. NPC is treated with systemic therapy, including radiotherapy and chemotherapy. Patients may experience adverse reactions in several systems, such as the gastrointestinal tract, blood, and skin, which may influence the patients’ final treatment strategy and, in severe cases, cause discontinuation of treatment or death.

Previous research has suggested that men and women have different incidence rates and severity of adverse effects during treatment; however, this has not been documented in NPC patients. Therefore, we conducted a retrospective study to analyze the impact of sex on treatment-related adverse effects and prognosis in NPC.

## Methods

### Participants

A total of 1,812 patients with NPC completed the entire course of treatment at Nanfang Hospital of the Southern Medical University between January 2004 and December 2015. Among those patients, 15 with non-primary NPC,6 with other malignant neoplasms, 57 who discontinued treatment, and 272 with missing medical records or who underwent radiotherapy alone were excluded. Finally, 1,462 patients were included in this study. This study was approved by the Medical Ethics Committee of Nanfang Hospital of the Southern Medical University (NFEC-201,710-K3).

### Study variables

The study variables included sex, age, TNM stage, clinical stage, chemotherapy regimen histological types, EBV DNA and grading for myelosuppression, gastrointestinal response, radiation laryngitis, and radiation dermatitis. Patients were further divided according to their age into the premenopausal (≤ 45 years), menopausal (46–54 years), and postmenopausal (≥ 55 years) groups.

The chemotherapy regimens were divided according to the specific treatment plan of patients with NPC defined as follows: low dose for concurrent chemotherapy alone; moderate dose for induction chemotherapy plus concurrent chemotherapy or concurrent chemotherapy plus adjuvant chemotherapy; and high dose for induction chemotherapy plus concurrent chemotherapy and adjuvant chemotherapy. The classification and evaluation of adverse reactions were performed by the senior attending physician or associate chief physician through analysis and consultation of the patients’ medical and examination records. The criteria for evaluation were based on the National Cancer Institute tumor treatment common adverse reaction classification version 5.0. The principle behind recording adverse reaction-related data was to record only the highest grade of adverse reactions.

### Treatment and follow-up

All patients received either intensity-modulated radiotherapy or conventional radiotherapy plus chemotherapy with induction, concurrent, and adjuvant chemotherapy regimens. The induction or adjuvant chemotherapy regimens included cisplatin in combination with 5-fluorouracil, cisplatin in combination with paclitaxel or cisplatin, and 5-fluorouracil in combination with paclitaxel every 3 weeks for two to three cycles. Synchronous chemotherapy with cisplatin was administered at weeks 1, 4, and 7 from the start of radiotherapy. During treatment, hydration, alkalinization, and gastric protection were provided prior to chemotherapy to prevent and reduce the incidence of adverse reactions.

Follow-ups were performed by checking routine outpatient medical records or by telephone consultation and were conducted every 3 months for the first 3 years, every 6 months for the next 2 years, and annually thereafter. The main prognostic indicators included overall survival (OS), progression-free survival (PFS), local recurrence-free survival (LRFS), and distant metastasis-free survival (DMFS), which were defined as the period between the beginning of treatment and death from any cause, the period between the beginning of treatment and occurrence of disease progression or death from any cause, the period between the start of treatment and first local recurrence, and the period between the start of treatment and occurrence of first distant metastasis, respectively.

### Statistical analysis

The covariate balance between women and men was examined by the t-test (continuous variable), χ^2^ test, or Fisher’s exact test (categorical variables), as appropriate, while nonparametric tests were used to examine the ordered categorical variables (such as clinical stage, T stage, and N stage). To compare the differences in adverse reactions, the patients were divided into groups according to age and chemotherapy regimens, and the difference in adverse reactions was evaluated by nonparametric tests. The relative risk ratio was calculated using a logistic regression model, and survival outcomes were analyzed using the Kaplan–Meier method. The log-rank test was used to compare the survival curves. The survival analysis including OS, PFS, LRFS, and DMFS was conducted at 5 years. All statistical analyses were performed using IBM SPSS Statistics version 26.0. Statistical *P*-values < 0.05 were considered significant.

## Results

### Patient characteristics

Between February 2004 and December 2015, 1,462 patients (male to female ratio 2.71 to 1) with NPC were included in the study.There were no significant differences between sexes in terms of T stage, N stage, clinical stage, pathological type, or chemotherapy dosage (Table 1).

**Table 1:** The characteristics for male and female patients

	Total	Male	Female	*P*
	*(N = 1462)*	*(N = 1068)*	*(N = 394)*	
**T-stage**				0.831
**T1**	257 (17.6%)	196 (18.4%)	61 (15.5%)	
**T2**	360 (24.6%)	256 (24%)	104 (26.4%)	
**T3**	283 (19.4%)	195 (18.3%)	88 (22.3%)	
**T4**	562 (38.4%)	421 (39.4%)	141 (35.8%)	
**N-stage**				0.480
**N0**	175 (12%)	136 (12.7%)	39 (9.9%)	
**N1**	470 (32.1%)	342 (32%)	128 (32.5%)	
**N2**	660 (45.1%)	471 (44.1%)	189 (48%)	
**N3**	157 (10.7%)	119 (11.1%)	38 (9.6%)	
**M-stage**				0.438
**M0**	1401 (95.8%)	1021 (95.6%)	380 (96.4%)	
**M1**	61 (4.2%)	47 (4.4%)	14 (3.6%)	
**Clinical stage** ^*^				0.182
**I**	51 (3.5%)	45 (4.2%)	8 (2%)	
**II**	249 (17%)	174 (16.3%)	73 (18.5%)	
**III**	467 (31.9%)	326 (30.5%)	143 (36.3%)	
**IV**	695 (47.5%)	523 (49%)	170 (43.1%)	
**Age**				0.038
**Mean**	45.86	46.25	44.89	
**SD**	11.208	11.199	11.186	
**Median**	45	46	44	
**Range**	12–77	13–77	12–76	
**Histologic type** ^**^				0.324
**1**	10 (0.7%)	7 (0.7%)	3 (0.8%)	
**2**	126 (8.6%)	85 (8%)	41 (10.4%)	
**3**	1326 (90.7%)	976 (91.4%)	350 (88.8%)	
**EBV-DNA**				0.068
**N**	373 (25.5%)	286 (26.8%)	87 (22.1%)	
**P**	1089 (74.5%)	782 (73.2%)	307 (77.9%)	
**Chemotherapy regimens** ^***^				0.138
**Low**	377 (25.8%)	270 (25.3%)	107 (27.2%)	
**Medium**	700 (47.9%)	504 (47.2%)	196 (49.7%)	
**High**	385 (26.3%)	294 (27.5%)	91 (23.1%)	

*TNM staging according to 7th Edition of the American Joint Committee on Cancer (7th AJCC). **Histologic type:based on the criteria of WHO histological type (2003): 1—Squamous-cell carcinomas;2—Differentiated non-keratinising carcinoma,;3—Undifferentiated non-keratinising carcinoma;4—basaloid squamous carcinoma

***AC = adjuvant chemotherapy;CC = concurrent chemotherapy;IC = induction chemotherapy;Low = CC;Medium = IC/ AC + CC;High = IC + CC + AC;

N: negative. P: positive

There were 680(male to female ratio 2.45 to 1), 473(male to female ratio 2.75 to 1), and 309(male to female ratio 3.35 to 1) patients in the premenopausal, menopausal, and postmenopausal groups, respectively. The T stage, N stage, clinical stage, and pathological type did not differ significantly between the age groups, There was a significant difference in the chemotherapy regimens between men and women in the premenopausal age group (P = 0.002), with high dose for a larger proportion of NPC in male patients (Table 2).

**Table 2:** The characteristics for male and female patients in three age groups

	Premenopausal age				Menopausal age			Postmenopausal age		
	male		female	*P*	male	female	*P*	male	female	*P*
	(*N* = 483)		(*N* = 197)		(*N* = 347)	(*N* = 126)		(*N* = 238)	(*N* = 71)	
**T-stage**				0.655			0.693		0.928	
**T1**	99(20.5%)		37(18.8%)		64(18.4%)	15(11.9%)		33(13.9%)	9(12.7%)	
**T2**	129(26.7%)		60(30.5%)		73(21%)	31(24.6%)		54(22.7%)	13(18.3%)	
**T3**	77(15.9%)		35(17.8%)		69(19.9%)	32(25.4%)		49(20.6%)	21(29.6%)	
**T4**	178(36.9%)		65 (33%)		141(40.6%)	48(38.1%)		102(42.9%)	28(39.4%)	
**N-stage**				0.807			0.783		0.22	
**N0**	49(10.1%)		16 (8.1%)		47 (13.5%)	13 (10.3%)		40(16.8%)	10(14.1%)	
**N1**	150(31.1%)		70 (35.5%)		105(30.3%)	38 (30.2%)		87(36.6%)	20(28.2%)	
**N2**	230(47.6%)		89(45.2%)		153(44.1%)	65(51.6%)		88(37%)	35(49.3%)	
**N3**	54(11.2%)		22(11.2%)		42(12.1%)	10(7.9%)		23(9.7%)	6(8.5%)	
**M-stage**				0.998			0.827		0.092	
**M0**	466(96.5%)		190 (96.4%)		330(95.1%)	119 (94.4%)		225(94.5%)	71(100%)	
**M1**	17(3.5%)		7(3.6%)		17(4.9%)	7(5.6%)		13(5.5%)		
**Clinical stage** ^*^				0.351			0.602		0.441	
**I**	17(3.5%)		2(1%)		18(5.2%)	5(4%)		10(4.2%)	1(1.4%)	
**II**	89(18.4%)		47(23.9%)		45(13%)	16(12.7%)		4016.8%)	10(14.1%)	
**III**	158(32.7%)		67(34%)		103(29.7%)	45 (35.7%)		65(27.3%)	31(43.7%)	
**IV**	219(45.3%)		81(41.1%)		181(52.2%)	60(47.6%)		123(51.7%)	29(40.8%)	
**Age**			0.459			0.526			0.788	
**Mean**	36.41		36.01		49.63	49.42		61.3	61.48	
**SD**	6.433		6.668		3.254	2.989		4.848	4.881	
**Median**	38		38		49	49		60	60	
**Range**	13–45		12–45		46–55	46–55		56–77	56–76	
**Histology** ^**^				0.436			0.347		0.238	
**1**	3(0.6%)		1(0.5%)		3(0.9%)	2(1.6%)		1(0.4%)		
**2**	39(8.1%)		22(11.2%)		31(8.9%)	10(7.9%)		15(6.3%)	9(12.7%)	
**3**	441(91.3%)		174(88.3%)		313(90.2%)	114(90.5%)		222(93.3%)	62(87.3%)	
**EBV**				0.218			0.96		0.671	
**N**	186(38.5%)		46(23.4%)		67(19.3%)	24(19%)		33(13.9%)	17(23.9%)	
**P**	297(61.5%)		151(76.6%)		280(80.7%)	102(81%)		205(86.1%)	54(76.1%)	
**Chemotherapy regimens** ^***^				0.002			0.247		0.537	
**Low**	93(19.3%)		55(27.9%)		85(24.5%)	24(19%)		92(38.7%)	28(39.4%)	
**Medium**		232(48%)	97(49.2%)		171(49.3%)	65(51.6%)		101(42.4%)	34(47.9%)	
**High**	158(32.7%)		45(22.8%)		91(26.2%)	37(29.4%)		45(18.9%)	9(12.7%)	

*TNM staging according to 7th Edition of the American Joint Committee on Cancer (7th AJCC). **Histologic type:based on the criteria of WHO histological type (2003): 1—Squamous-cell carcinomas;2—Differentiated non-keratinising carcinoma,;3—Undifferentiated non-keratinising carcinoma;4—basaloid squamous carcinoma. ***AC = adjuvant chemotherapy;CC = concurrent chemotherapy;IC = induction chemotherapy. Low = CC;Medium = IC/ AC + CC;High = IC + CC + AC;

There were 377(male to female ratio 2.52 to 1), 700 (male to female ratio 2.57 to 1)and 385(male to female ratio 3.23 to 1) patients in the low dose, moderate dose, and high dose, respectively.There was a significant difference in the pathological findings between men and women in the low chemotherapy dosage group (P = 0.027), with non-keratinized undifferentiated carcinoma accounting for a larger proportion of NPC in male patients (Table 3).

**Table 3:** The characteristics for male and female patients in three chemotherapy regimens

	Low			Medium			High		
	male	female	*P*	male	female	*P*	male	female	*P*
	(*N* = 270)	(*N* = 107)		(*N* = 504)	(*N* = 196)		(*N* = 294)	(*N* = 91)	
**T-stage**			0.861			0.572		0.421	
**T1**	55 (20.4%)	20 (18.7%)		94 (18.7%)	28 (14.3%)		47 (16%)	13 (14.3%)	
**T2**	78 (28.9%)	33 (30.8%)		125 (24.9%)	52 (26.5%)		53 (18%)	19 (20.9%)	
**T3**	39 (14.4%)	19 (17.8%)		96 (18.9%)	44 (22.4%)		60 (20.4%)	25 (27.5%)	
**T4**	98 (36.3%)	35 (32.7%)		189 (37.6%)	72 (36.7%)		134 (45.6%)	34 (37.4%)	
**N-stage**		0.498	0.498			0.577		0.672	
**N0**	65 (24.1%)	22 (20.6%)		49 (9.7%)	12 (6.1%)		22 (7.5%)	5 (5.5%)	
**N1**	97 (35.9%)	37 (34.6%)		165 (32.7%)	66 (33.7%)		80 (27.2%)	25 (27.5%)	
**N2**	83 (30.7%)	41 (38.3%)		229 (45.4%)	98 (50%)		159 (54.1%)	50 (54.9%)	
**N3**	25 (9.3%)	7 (6.5%)		61 (12.1%)	20 (10.2%)		33 (11.2%)	11 (12.1%)	
**Clinical stage**			0.739			0.5		0.369	
**I**	21 (7.8%)	4 (3.7%)		20 (4%)	3 (1.5%)		4 (1.4%)	1 (1.1%)	
**II**	69 (25.6%)	26 (24.3%)		73 (14.5%)	35 (17.9%)		32 (10.9%)	12 (13.2%)	
**III**	60 (22.2%)	39 (36.4%)		166 (32.9%)	70 (35.7%)		100 (34%)	34 (37.4%)	
**IV**	120 (44.4%)	38 (35.5%)		245 (48.6%)	88 (44.9%)		158 (53.7%)	44 (48.4%)	
**Age**		0.01			0.496			0.875	
**Mean**	50.32	45.89		45.70	45.09		43.47	43.27	
**SD**	11.638	13.220		10.663	10.536		10.659	9.806	
**Median**	50	43		45	45		44	45	
**Range**	24–77	14–76		14–74	12–72		13–70	19–63	
**EBV**			0.407			0.053		0.075	
**N**	67 (24.8%)	31 (29%)		133 (26.4%)	38 (19.4%)		86 (29.3%)	18 (19.8%)	
**P**	203 (75.2%)	76 (71%)		371 (73.6%)	158(80.6%)		208 (70.7%)	73 (80.2%)	
**Histology**			0.027			0.833		0.55	
**1**		2 (1.9%)		4 (0.8%)	1 (0.5%)		3 (1%)		
**2**	31 (11.5%)	18 (16.8%)		36 (7.1%)	16 (8.2%)		18 (6.1%)	7 (7.7%)	
**3**	239 (88.5%)	87 (81.3%)		464 (92.1%)	179(91.3%)		273 (92.9%)	84 (92.3%)	

*TNM staging according to 7th Edition of the American Joint Committee on Cancer (7th AJCC). **Histologic type:based on the criteria of WHO histological type (2003): 1—Squamous-cell carcinomas;2—Differentiated non-keratinising carcinoma,;3—Undifferentiated non-keratinising carcinoma;4—basaloid squamous carcinoma. AC = adjuvant chemotherapy;CC = concurrent chemotherapy;IC = induction chemotherapy. Low = CC;Medium = IC/ AC + CC;High = IC + CC + AC;

### Survival analysis

**Figs. 1:**
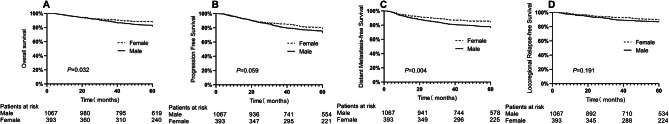
Survival curves stratified by sex for patients in the entire cohort

Kaplan–Meier survival analysis was performed for 1,462 patients who were divided into groups according to sex (Fig. 1A–D), and the results showed that OS (81.5% vs. 87.1%, P = 0.032), LRFS (86.7% vs. 89.1%, P = 0.191), PFS (72.6% vs. 78.3%, P = 0.059), DMFS (76.2% vs. 83.9%, P = 0.004), and prognosis were better in female patients than in male patients at all survival endpoints, with OS and DMFS being significantly different between sexes.

**Figs. 2:**
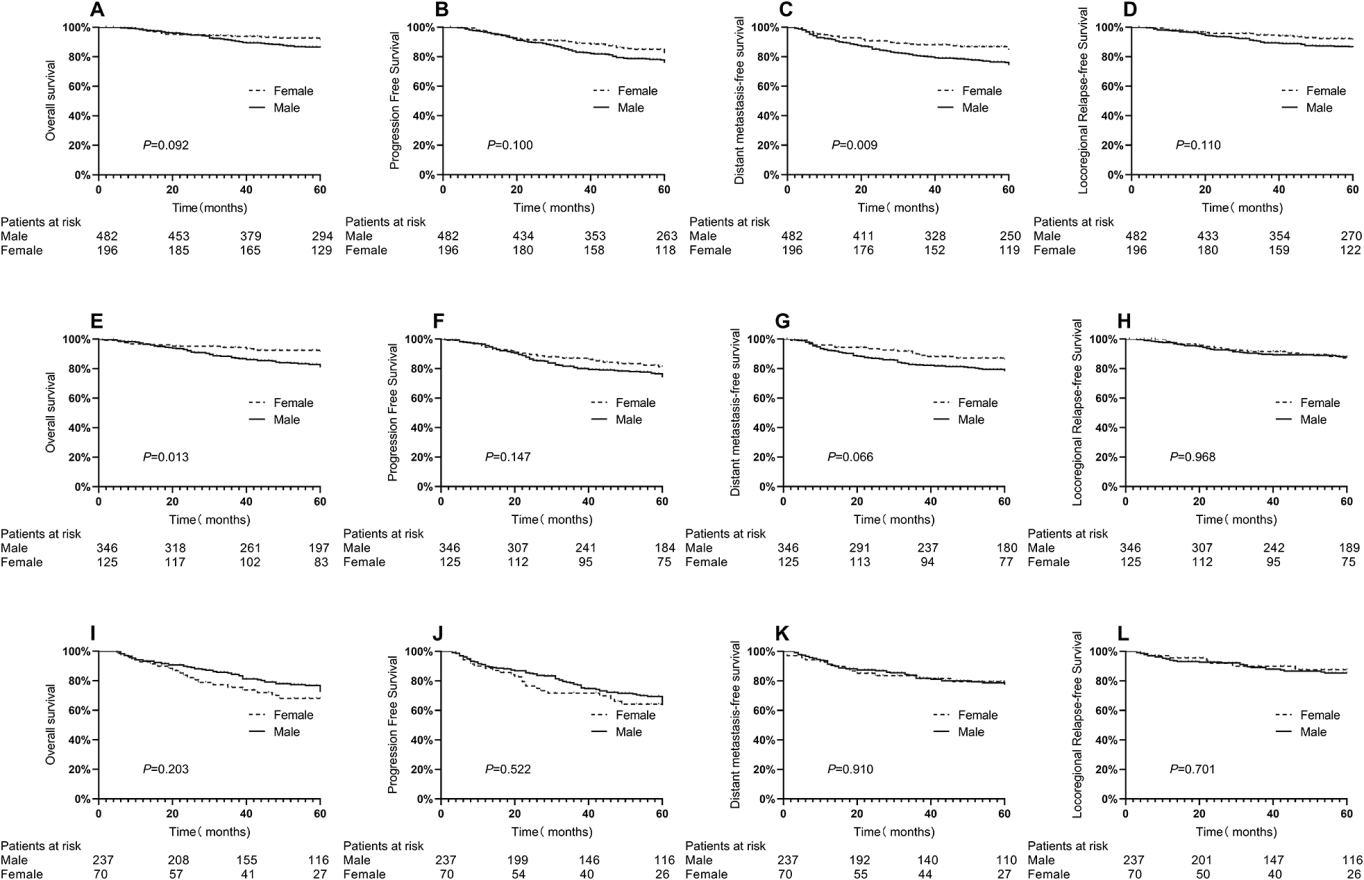
Survival curves stratified by sex and age for patients in the entire cohort. D: premenopausal; E-H: menopausal; I-L: postmenopausal

The analysis of survival between men and women in each age group after stratification showed that compared with women, men had worse survival at all end points compared to women in the premenopausal group (Fig. 2A–D). Additionally, the DMFS was significantly different between men and women in the premenopausal group (74.3% vs. 84.2%, P = 0.009). Meanwhile, OS (80.9% vs. 91.4%, P = 0.092),PFS(75.9% vs. 81.8%, P = 0.100) and LRFS (86.4% vs. 91.0%, P = 0.110) showed that prognosis in women tended to be better than that in men, albeit without significance. Additionally, in the menopausal age group (Fig. 2E–H), OS (80.9% vs. 91.4%, P = 0.013), DMFS (78.2% vs. 86.0%, P = 0.066), and PFS (74.0% vs. 81.2%, P = 0.147) were better in women than in men. In contrast, there was no significant difference in survival between men and women in the postmenopausal group as shown by the following: OS (72.4% vs. 67.1%, P = 0.203), PFS (63.4% vs. 63.3%, P = 0.434), DMFS (77.3% vs. 79.5%, P = 0.910), and LRFS (85.4% vs. 87.2%, P = 0.701) (Fig. 2I–L). Based on the results, the prognoses of premenopausal and menopausal women were better than those of men, whereas those of postmenopausal women were not significantly different from those of men.

**Figs. 3:**
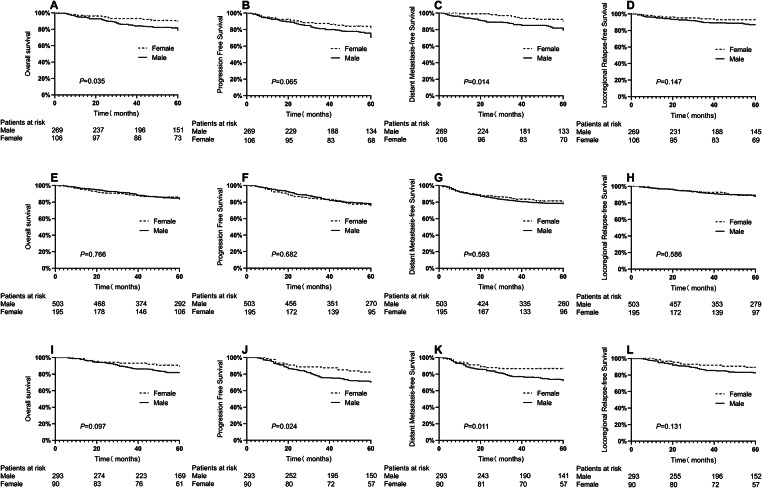
Survival curves stratified by sex and chemotherapy regimens for patients in the entire cohort. A-D: low dose; E-H: moderate dose; I-L: high dose

When patients were stratified according to chemotherapy regimens, in the low-dose group, OS (78.5% vs. 88.8%, P = 0.035) and DMFS (78.8% vs. 88.9%, P = 0.014) were significantly better in women than in men (Fig. 3A–D), whereas the LRFS (87.0% vs. 92.9%, P = 0.147) and PFS (69.9% vs. 81.0%, P = 0.065) tended to be better in women than in men, albeit without significance. In the moderate-dose group, the survival endpoints were not significantly different between men and women (Fig. 3E–H). In the high-dose group (Fig. 3I–L), the DMFS (71.7% vs. 86.7%, P = 0.011) and PFS (69.8% vs. 82.5%, P = 0.024) were significantly better in women than in men, while the OS (81.5% vs. 89.2%, P = 0.097) and LRFS (82.1% vs. 89.1%, P = 0.131) tended to be better in women than in men, albeit without significance.

### Adverse reactions

Statistical analysis (Table 4) was performed after the patients were grouped by sex, and the results showed that bone marrow suppression (U = 5.789; P < 0.001) and gastrointestinal reactions (U = 4.407 P < 0.001) were more severe in women than in men.

**Table 4:** Difference of adverse reactions

	Total	Male	Female	*P*
**Myelosuppression**				<0.001
**0**	279 (19.1%)	230 (21.5%)	49 (12.4%)	
**1**	312 (21.3%)	245 (22.9%)	67 (17%)	
**2**	475 (32.5%)	341 (31.9%)	134 (34%)	
**3**	251 (17.2%)	156 (14.6%)	95 (24.1%)	
**4**	145 (9.9%)	96 (9%)	49 (12.4%)	
**Gastrointestinal reaction**				<0.001
**0**	173 (11.8%)	129 (12.1%)	44 (11.2%)	
**1**	737 (50.4%)	569 (53.3%)	168 (42.6%)	
**2**	459 (31.4%)	322 (30.1%)	137 (34.8%)	
**3**	92 (6.3%)	47 (4.4%)	45 (11.4%)	
**4**	1 (0.1%)	1 (0.1%)		
**Radiation laryngitis**				0.904
**0**	64 (4.4%)	53 (5%)	11 (2.8%)	
**1**	640 (43.8%)	456 (42.7%)	184 (46.7%)	
**2**	600 (41%)	449 (42%)	151 (38.3%)	
**3**	137 (9.4%)	93 (8.7%)	44 (11.2%)	
**4**	21 (1.4%)	17 (1.6%)	4 (1%)	
**Radiation dermatitis**				0.455
**0**	83 (5.7%)	62 (5.8%)	21 (5.3%)	
**1**	862 (59%)	624 (58.4%)	238 (60.4%)	
**2**	390 (26.7%)	279 (26.1%)	111 (28.2%)	
**3**	127 (8.7%)	103 (9.6%)	24 (6.1%)	

The criteria for evaluation were based on the NCI tumor treatment common adverse reaction classification version 5.0

After classifying the patients based on age, a comparison between sexes showed that the severity of myelosuppression was greater in women than in men at all ages, and that the severity of gastrointestinal reactions in women in the premenopausal and menopausal age groups were greater than those in men (Table 5). In contrast, there was no statistically significant difference in the gastrointestinal reactions between men and postmenopausal women. Additionally, there were no statistically significant differences in radiotherapy-related adverse effects between sexes at all ages.

**Table 5:** Difference of adverse reactions after grouping according to age

	Premenopausal age			Menopausal age			Postmenopausal age			
	**male**	**female**	***P***	**male**	**female**	***P***	**male**	**female**	*P*	
**Myelosuppression**			0.011			0.001		0.002		
**0**	79 (16.4%)	25 (12.7%)		92 (26.5%)	15 (11.9%)		59 (24.8%)	9 (12.7%)		
**1**	123 (25.5%)	37 (18.8%)		76 (21.9%)	17 (13.5%)		46 (19.3%)	13 (18.3%)		
**2**	153 (31.7%)	65 (33%)		112 (32.3%)	51 (40.5%)		76 (31.9%)	18 (25.4%)		
**3**	76 (15.7%)	46 (23.4%)		47 (13.5%)	31 (24.6%)		33 (13.9%)	18 (25.4%)		
**4**	52 (10.8%)	24 (12.2%)		20 (5.8%)	12 (9.5%)		24 (10.1%)	13 (18.3%)		
**Gastrointestinal reaction**			0.001			0.008		0.593		
**0**	49 (10.1%)	15 (7.6%)		48 (13.8%)	17 (13.5%)		32 (13.4%)	12 (16.9%)		
**1**	266 (55.1%)	88 (44.7%)		185 (53.3%)	52 (41.3%)		118 (49.6%)	28 (39.4%)		
**2**	140 (29%)	73 (37.1%)		104 (30%)	37 (29.4%)		78 (32.8%)	27 (38%)		
**3**	28 (5.8%)	21 (10.7%)		9 (2.6%)	20 (15.9%)		10 (4.2%)	4(5.6%)		
**4**				1 (0.3%)						
**Radiation laryngitis**			0.608			0.826		0.973		
**0**	21 (4.3%)	6 (3%)		22 (6.3%)	3 (2.4%)		10 (4.2%)	2(2.8%)		
**1**	205 (42.4%)	95 (48.2%)		151 (43.5%)	58 (46%)		100 (42%)	31 (43.7%)		
**2**	213 (44.1%)	73 (37.1%)		138 (39.8%)	50 (39.7%)		98 (41.2%)	28 (39.4%)		
**3**	38 (7.9%)	20 (10.2%)		29 (8.4%)	15 (11.9%)		26 (10.9%)	9 (12.7%)		
**4**	6 (1.2%)	3 (1.5%)		7 (2%)			4 (1.7%)	1(1.4%)		
**Radiation dermatitis**			0.97			0.961		0.058		
**0**	32 (6.6%)	11 (5.6%)		20 (5.8%)	5 (4%)		10 (4.2%)	5 (7%)		
**1**	292 (60.5%)	119 (60.4%)		201 (57.9%)	74 (58.7%)		131 (55%)	45 (63.4%)		
**2**	114 (23.6%)	53 (26.9%)		93 (26.8%)	41 (32.5%)		72 (30.3%)	17 (23.9%)		
**3**	45 (9.3%)	14 (7.1%)		33 (9.5%)	6 (4.8%)		25 (10.5%)	4 (5.6%)		

The criteria for evaluation were based on the NCI tumor treatment common adverse reaction classification version 5.0

In patients who underwent similar chemotherapy regimens, bone marrow suppression and gastrointestinal reactions were more severe in women than in men (Table 6). Although there was no statistically significant difference in the severity of adverse reactions between men and women in the high-dose chemotherapy group, the mean rank of women was higher than that of men (189.10 vs. 205.62, P = 0.176). There were no significant differences in radiotherapy-related adverse reactions between sexes.

**Table 6:** Difference of adverse reactions after grouping according to chemotherapy regimens

	Low			Medium			High		
	male	female	*P*	male	female	*P*	male	female	*P*
**Myelosuppression**			<0.001			<0.001		0.009	
**0**	97 (35.9%)	17 (15.9%)		66 (13.1%)	11 (5.6%)		28 (9.5%)	8 (8.8%)	
**1**	55 (20.4%)	21 (19.6%)		134(26.6%)	33(16.8%)		75 (25.5%)	13(14.3%)	
**2**	65 (24.1%)	32 (29.9%)		174(34.5%)	80(40.8%)		112(38.1%)	29(31.9%)	
**3**	28 (10.4%)	19 (17.8%)		81 (16.1%)	52 (26.5%)		54 (18.4%)	29 (31.9%)	
**4**	25 (9.3%)	18 (16.8%)		49 (9.7%)	20 (10.2%)		25 (8.5%)	12 (13.2%)	
**Gastrointestinal reaction**			<0.001			0.017		0.176	
**0**	31 (11.5%)	13 (12.1%)		68 (13.5%)	20(10.2%)		30 (10.2%)	11(12.1%)	
**1**	152(56.3%)	38 (35.5%)		263(52.2%)	92(46.9%)		154(52.4%)	38(41.8%)	
**2**	79 (29.3%)	40 (37.4%)		147(29.2%)	65(33.2%)		96 (32.7%)	32(35.2%)	
**3**	8 (3%)	16 (15%)		25 (5%)	19 (9.7%)		14 (4.8%)	10 (11%)	
**4**				1 (0.2%)					
**Radiation laryngitis**			0.672			0.826		0.766	
**0**	15 (5.7%)	3 (2.8%)		25 (5%)	3 (1.6%)		13 (4.4%)	5 (5.5%)	
**1**	107(40.4%)	48 (44.9%)		197(39.2%)	88(45.6%)		151(51.4%)	48(52.7%)	
**2**	116(43.8%)	50 (46.7%)		223(44.4%)	73(37.8%)		104(35.4%)	28 (30.8%)	
**3**	25 (9.4%)	6 (5.6%)		47 (9.4%)	26 (13.5%)		21 (7.1%)	9 (9.9%)	
**4**	2 (0.8%)			10 (2%)	3 (1.6%)		5 (1.7%)	1 (1.1%)	
**Radiation dermatitis**			0.329			0.345		0.595	
**0**	16 (5.9%)	7 (6.5%)		26 (7%)	11 (7.5%)		20 (6.8%)	3 (3.3%)	
**1**	156 (58%)	66 (61.7%)		209(56.6%)	87(59.2%)		182(61.9%)	56(62.2%)	
**2**	72 (26.8%)	29 (27.1%)		84 (22.8%)	35 (23.8%)		65 (22.1%)	28 (31.1%)	
**3**	25 (9.3%)	5 (4.7%)		50 (13.6%)	14 (9.5%)		27 (9.2%)	3 (3.3%)	

The criteria for evaluation were based on the NCI tumor treatment common adverse reaction classification version 5.0

Due to significant statistical differences in myelosuppression and gastrointestinal reactions, logistic regression analysis was performed to adjust the comparisons between sexes by the following baseline characteristics: chemotherapy regimens, and clinical staging (Table 7). Grade 3–4 myelosuppression and gastrointestinal reactions were defined as the occurrence of severe adverse events, and sex was found to be an important predictor of severe myelosuppression and gastrointestinal reactions.

**Table 7:** Logistic Regression: severe adverse or not

	Gastrointestinal reaction				Myelosuppression			
	*P*	OR	95%CI		*P*	OR	95%CI	
Clinical stage	0.686	1.055	0.815	1.366	0.002	1.255	1.084	1.453
chemotherapy	0.883	0.978	0.723	1.322	0.033	1.196	1.014	1.41
gender(male)	0.001	2.696	1.761	4.128	<0.001	1.904	1.18	2.449

## Discussion

Several studies have examined the impact of sex on the prognosis of many non-reproductive malignancies, and the results showed that under similar treatment regimens, women tended to have better prognosis than men [14–18]. In two recent studies on NPC, researchers used statistical analysis to eliminate the effects of smoking, drinking status, body mass index, and various indicators (T stage, N stage, clinical stage) associated with diagnosis delay and concluded that the prognostic advantage of female patients over male patients was age-dependent. In this study, the prognostic indicators were analyzed after grouping the patients by age according to the criteria of the classic review [19]. Our results indicate that the prognosis of female patients was better than that of male patients during the premenopausal and menopausal periods, but this prognostic advantage was not observed after menopause, suggesting that sex hormones may play a role in the prognosis of patients with NPC. However, probably due to insufficient sample size, the statistical difference in prognosis by gender was not significant.

In clinical practice, we often find that treatment-related adverse effects in NPC seem to be more severe in women than in men. In this study, by analyzing the adverse reactions of all patients and further stratifying them by age group and treatment regimen, the results showed that the severity of myelosuppression and the risk of severe (grade 3–4) adverse reactions during treatment was significantly greater in women than in men. The incidence of toxicity with cytotoxic drug use was reported to be higher in women [20]. Previous studies have shown that paclitaxel not only saturates the peripheral compartment at lower plasma levels in women than in men (0.83 vs. 1.74 µmol/L) but also has lower plasma elimination, resulting in a longer duration of exposure [21]. Based on body surface area (BSA), maximum plasma concentrations were higher in women even after dose adjustment, which can also be attributed to lower CYP3A4 enzyme activity and P-glycoprotein levels in women than in men. Hence, fewer doses of paclitaxel are metabolized by CYP3A4 and excreted into the bile via P-glycoprotein in women, resulting in a longer half-life, which increases the time to exceed toxicological threshold concentrations in women [22]. The pharmacokinetics of cisplatin also differ significantly between men and women which may be due to changes in the expression of metabolic enzymes in the kidneys and liver [23]. For example, the metabolism of cisplatin is positively correlated with glutathione S-transferase activity, which affects the uptake and retention of the drug in the kidneys and liver [24]. In women, cisplatin-induced toxicity may be caused by higher glutathione S-transferase activity, which leads to longer biological half-lives and higher drug retention in the target organs [25]. In another studies, women had higher blood levels and longer half-lives of 5-fluorouracil under the BSA-based dosing regimen [26, 27]. In addition, the main protein groups responsible for binding drugs in human plasma are influenced by the concentration of sex hormones; thus, plasma drug binding is clearly influenced by sex, which in turn affects the drugs’ pharmacokinetics [28]. In chemotherapy for patients with NPC, sex-differentiated expression levels of metabolic enzymes and transport proteins in the liver and kidneys result in altered pharmacokinetics of most anticancer drugs [29]. Regarding chemotherapy drugs, blood concentration and half-lives of drugs are higher in women under the same BSA-based dosing regimen. However, the recommended doses of drugs are usually determined in male-dominated phase I and II trials without considering the potential impact of sex on the optimal dosing [30, 31]. The maximum tolerated dose of certain drugs may be lower in women, and administration of standard doses may result in increased blood drug concentrations and toxicity. Hence, chemotherapy regimens based on BSA and body weight are being increasingly questioned in existing cancer treatments, and researchers are actively exploring more rational dosing regimens such as those based on fat-free weight [32, 33]. In addition, there is a dose-response relationship for chemotherapeutic agents [20, 23], and previous studies have found a positive correlation between higher drug response rates and longer survival in female tumour patients [34, 35]. In contrast, the lower toxicity rate in men may be explained by relative underdosage, which may contribute to their poorer prognosis.

Another reason for the greater severity of gastrointestinal reactions in women than in men in this study was the difference in the response to antiemetic drugs, e.g., gastrodia, 5-hydroxytryptamine receptor modulators, or neurokinin-1 antagonists were less effective in women than in men [36–38]. These drugs used during chemotherapy also affect the difference in the gastrointestinal reactions between men and women. However, the incidence of gastrointestinal reactions was not different between men and women of all ages in the high-dose group, or between men and postmenopausal women in all dose groups. This may be due to the reduced dose of chemotherapy drugs and the lack of sensitivity to gastrointestinal symptoms, such as nausea and vomiting, in older women.

Radiation dermatitis and laryngitis are caused by radiation therapy. A previous study on radiation therapy for head and neck tumors concluded that sex did not contribute to the differences in Karnofsky Score, mucositis, and presence of thrush and dermatitis scores [39, 40]. In this study, no differences were found between men and women, either between age groups or between chemotherapy doses. This finding is consistent with the results of previous studies. Another study also showed that patients experienced radiotherapy-related adverse effects mainly related to the mode of radiotherapy and nursing, and that the relationship with sex was unclear [41].

This was a single-center retrospective study with a relatively small size after subgrouping. Data collection and grading were mainly derived from medical records, which can be biased by subjective factors and source of patients. Radiotherapy was mostly intensity-modulated radiotherapy, and as this was a retrospective study, a majority of detailed radiotherapy data could not be acquired. Furthermore, in this study, only the highest level of adverse reactions during treatment was recorded and not the frequency of occurrence. The interpretation of radiotherapy-related adverse reactions between sexes is incomprehensive, and further research is required.

## Conclusion

Chemotherapy-related adverse effects in the treatment of NPC were more severe in women than in men, but the overall prognosis was better in women than in men. To create more individualized treatment strategies, establishing sex-related treatment and observation criteria may benefit patients.

## Data Availability

The datasets generated and analyzed during the current study are not publicly available because the dataset will be further studied to publish other works but are available from the corresponding author on reasonable request.

## Abbreviations

BSA: Body surface area
DMFS: Distant metastasis-free survival
LRFS: Local recurrence-free survival
OS: Overall survival
PFS: Progression-free survival
